# Implementation of a Rapid RT-LAMP Saliva-Based SARS-CoV-2 Testing Program in the Workplace

**DOI:** 10.3390/diagnostics12020474

**Published:** 2022-02-12

**Authors:** Bradley W. M. Cook, Kaitlyn Kobasa, Marielou Tamayo, Natasha Theriault, Diane Gordon Pappas, Steven S. Theriault

**Affiliations:** 1 Cytophage Technologies Inc., Winnipeg, MB R3Y 1G4, Canada; kaitlyn@cytophage.com (K.K.); marielou@cytophage.com (M.T.); natasha@cytophage.com (N.T.); diane@cytophage.com (D.G.P.); steven@cytophage.com (S.S.T.); 2 Department of Microbiology, The University of Manitoba, Winnipeg, MB R3T 2N2, Canada

**Keywords:** SARS-CoV-2, COVID-19, rapid molecular testing, RT-LAMP, diagnostics, occupational health and safety, group testing, self-administration test

## Abstract

Rising SARS-CoV-2 cases, testing delays, and the risk of pre-symptomatic and asymptomatic transmission provided the impetus for an in-house rapid testing program. Employees and their household contacts were encouraged to self-collect saliva samples that were pooled for routine testing using an established colorimetric reverse transcription loop-mediated isothermal amplification (RT-LAMP) assay. In brief, individual or a maximum of four saliva samples were pooled and heat-inactivated to render microorganisms, especially SARS-CoV-2, non-infectious prior to being added to RT-LAMP assay tubes containing either the human sample control gene, RNase P, or a region of the SARS-CoV-2 gene, ORF1ab. During the second wave of SARS-CoV-2 infections in November 2020, two samples from an employee and a member of their household tested positive via RT-LAMP within two days of each other. A delayed clinical qRT-PCR test confirmation of both individuals 5 days later underscored the power of routine rapid testing with within-the-hour turnaround times. Workplace rapid testing programs using RT-LAMP are flexible in their design, have a reduced cost compared to qRT-PCR, may involve non-invasive self-saliva collection for increased safety for the testing personnel, and can be performed with minimal training.

## 1. Introduction

**Figure 1:**
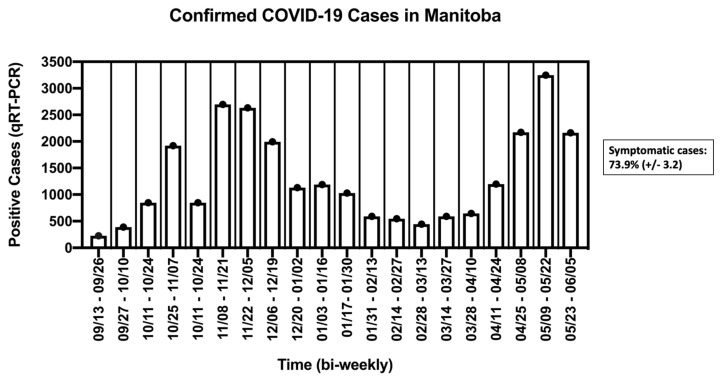
Epidemiological data of qRT-PCR positive cases in Manitoba from 13 September 2020 to 5 June 2021. Data were compiled from Manitoba’s Provincial COVID-19 Surveillance System (https://www.gov.mb.ca/health/publichealth/surveillance/covid-19/index.html. Accessed on 30 November 2021) presented as a rolling average of two-week case counts. Every two-week interval included in the analysis reported the percentage of individuals that were symptomatic at the time of testing (73.9% (±3.2)). An overall average was calculated from each two-week interval.

Quantitative reverse-transcription-polymerase chain reaction (qRT-PCR) is recognized as the gold standard diagnostic test to determine if an individual is infected with SARS-CoV-2. Samples are obtained from a nasopharyngeal swab or the less invasive, nasal swab. This test strategy requires costly and sometimes supply limited consumables, hours of skilled labor, and expensive machinery for testing and analysis [1]. All of which are typically not available outside of clinical settings or an established academic laboratory. During the summer of 2020 in Manitoba, Canada, the demand for qRT-PCR testing led to processing delays and extended turnaround times of up to several days for results [2,3,4]. Long turnaround times exacerbate community spread as testing is voluntary and positive results would be skewed towards symptomatic individuals [5]; in Manitoba, more than two-thirds of those submitted for testing were symptomatic (summarized in Figure 1). This bias meant that 20–59% of infected individuals that were either pre-symptomatic or asymptomatic were unobserved by public health authorities, and may have led to further transmission [6,7]. The public health impact of such delays necessitates the use of cheap, easy-to-manufacture, scalable, and affordable rapid testing options that can aid in the ability to pre-screen for potentially contagious individuals. Rapid tests can be performed without highly trained personnel or complicated machinery (end point is detected visually), and are amenable for rapid daily-use to detect contagious individuals [1]. Reverse transcription loop-mediated isothermal amplification (RT-LAMP) is a rapid testing technique that amplifies the target gene(s), resulting in the continual amplification of stem-loop DNA structures at a single temperature [8]. While many RT-LAMP assays and detection variations exist [9], we utilized a previously optimized assay by Yang et al., 2020, for the SARS-CoV-2 screening program, with acidic saliva samples and a limit of detection (LOD) of 200 virions/µL (Ct of ~30) [10]. We hypothesized that routine RT-LAMP testing would significantly decrease the opportunity for the workplace transmission of SARS-CoV-2. We endeavored on a 9 month-long prescreening program for laboratory staff and, on occasion, included household contacts.

## 2. Materials and Methods

### 2.1. Study Design

**Figure 2:**
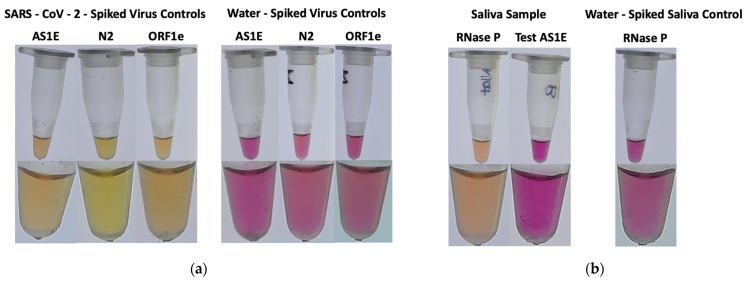
Expected RT-LAMP reaction results. (**a**) Control saliva-sample tubes were mixed 1:1 with a stabilization buffer and nuclease-free water, spiked with 2 µL of heat-inactivated SARS-CoV-2 or with 2 µL nuclease-free water, as indicated, and incubated for 10 min at 95 °C. Four µL of respective mixtures were added to RT-LAMP reactions with SARS-CoV-2 primer sets (AS1E, N2, or ORF1e), and were incubated for 30 min at 65 °C followed by 2 min at 80 °C. Images were taken on a lightbox with 4× (top panel) and 10× (bottom panel) magnification. (**b**) Saliva-sample tubes were mixed 1:1 with a stabilization buffer and saliva or nuclease-free water, as indicated, and incubated for 10 min at 95 °C. RT-LAMP reactions with the RNase P primer set were prepared, and 4 µL of the respective mixtures were added and incubated for 30 min at 65 °C followed by 2 min at 80 °C. Images were taken on a lightbox with 4× (top panel) and 10× (bottom panel) magnification. A negative amplification of the gene target resulted in the solution to remain pink (basic pH), whereas the target gene amplification results in a color change from pink to yellow (acidic pH).

The premise of the testing regime was to prevent or reduce the likelihood of workplace transmission, especially during times when a high demand would result in qRT-PCR testing/results delays. Employees were asked to remain home if they or family members were sick, and were asked for voluntary collection of a contact-less saliva sample for testing. While at the workplace, non-pharmaceutical interventions such as physical distancing, masking, and frequent handwashing were adhered to, in compliance with local public health orders [11]. A three-testing-days-a-week (generally Monday, Wednesday, and Friday, including Tuesday or Thursday in lieu of an observed holiday) plan was implemented to voluntarily test employees prior to beginning work. Outlier samples from friends and family were sporadically accepted. Employees were given a unique identification number at random draw, this number was used throughout the testing period, then two to four employee samples were pooled as a group with a letter designation. Outlier samples were given a unique number and were either tested individually or were pooled with other outliers to comprise a unique and separate letter group. The program commenced on 16 September 2020 and concluded on 31 May 2021. Positive group pools were disaggregated and individually retested (referred to as reflex testing) with all three SARS-CoV-2 RT-LAMP primer sets to determine a true positive. Test-positive individuals were asked to leave the workplace and follow the Manitoba Public Health Guidelines regarding isolation and visit an official COVID-19 Testing Centre for qRT-PCR testing. Pending qRT-PCR confirmation of a positive result, employees adhered to return to work policies set by Manitoba Public Health [12]. Figure 2 demonstrates the expected results for the screening program.

### 2.2. Saliva-Sample Preparation

Sample tube master mix preparation. Sample tubes were prepared with 2× Stabilization buffer, as described previously [10]. Briefly, stock containing 5 mM (717 mg) TCEP-HCl (Gold Bio #TCEP10, Cedarlane Labs, Burlington, Canada), 2 mM (2 mL) 0.5 M pH8 EDTA (#324506-100ML, Millipore-Sigma, Oakville, Canada), 29 mM (1.45 mL) 10 M NaOH pellets (#S8045-500G, Millipore-Sigma, Oakville, Canada) previously dissolved in nuclease-free water, 100 µg/mL (50mg) recombinant PCR-grade Proteinase K (Roche #3115879001, Cedarlane Labs, Burlington, Canada), and then the solution was brought up to 500 mL with nuclease-free water (10220-404, VWR, Mississauga, Canada). One mL aliquots of 2× Stabilization buffer were deposited into nuclease-free, were graduated in 5 mL screw-cap sample tubes (#10002-738, VWR, Mississauga, Ontario, Canada), and were stored at 4 °C until use. 

Saliva-sampling and processing. On scheduled testing days, employees were asked to abstain from consuming any food, beverages, or brushing teeth half an hour prior to producing a sample. The employees would independently remove a sample tube, containing 1 mL 2× Stabilization buffer kept at 4 °C, and provide 1 mL of fresh saliva from an isolated location. Then, the tubes were shaken vigorously for 10 s, labeled with their respective unique number identifier, and the exterior was decontaminated with 70% ethanol for 60 s at room temperature, and then stored at 4 °C in a designated location [13,14]. The samples were generally processed for the LAMP assay within a few hours; however, samples could be stored for up to 4 days [10]. Saliva-sample processing. Sample tubes were vortexed briefly, and then heat-inactivated in water baths (thermometer-verified) for 10 min at 95 °C. Tubes were then plunged in ice and transferred into a biological safety cabinet for the LAMP assay preparation.

### 2.3. Colorimetric Reverse Transcription Loop-Mediated Isothermal Amplification (RT-LAMP) Assay

Primers. 

Desalted primer sets were fabricated by IDT (Coralville, Iowa, USA), according to the sequences provided previously by Yang et al. [10]. Primer sets were diluted and mixed to make 10x concentration of primers targeting different regions in the SARS-CoV-2 genome: the *ORF1ab* gene (A1SE and ORF1e) or the *N* gene (N2), and a control for human saliva (RNaseP) that detects the *RNase P* gene. A1SE, ORF1e, N2, and RNaseP primer sets contained: FIB and BIP (16 µM), LF and LB (4 µM), and F3 and B3 (2 µM) primers, except LF was omitted from N2. The sequences are provided in Supplementary Table S1.

RT-LAMP assay master mix. Reactions were prepared as independent master mixes, as described previously [10]. Briefly, 4 µL of nuclease-free water, 10 µL Warmstart colorimetric LAMP 2× master mix (#M1800L, New England Biolabs, Whitby, Canada), and 2 µL respective 10× primer set mix (either AS1E, ORF1e, N2 or RNase P) were combined and aliquoted into PCR tubes. For routine testing, only the AS1E reactions were used for randomly pooled saliva samples and virus controls, and only one group at random was tested for RNAse P. For test reactions, either 4 µL from an individual’s saliva-stabilization buffer sample or an equal division of up to four saliva-stabilization samples was added, but it never exceeded four pooled samples (1 µL each) per group. Negative control and SARS-CoV-2 positive control reactions were prepared similarly, except 4 µL nuclease-free water in 2× stabilization buffer or 4 µL of SARS-CoV-2 control virus, were added instead of the saliva. The 20 µL reactions were gently vortex and incubated in a thermal cycler at 65 °C for 30 min, but never exceeded 35 min, followed by heat inactivation for 2 min at 80 °C in order to preserve color and prevent further unwanted amplification. Reaction tubes were visually inspected for color-change due to pH changes during amplification of viral RNA (basic pH-pink color negative and acidic pH-yellow color positive) and documented immediately after heat inactivation. In the event of a positive test, the group was split into individual samples and tested with all primer sets, along with the appropriate negative and virus controls.

SARS-CoV-2 virus controls. Two microliters from a stock of 1.6 × 10^5^ TCID_50_/mL (1.8 × 10^8^ RNA genome copies/mL) heat-inactivated SARS-CoV-2 (2019-nCoV/USA/WA1/2020, ATCC VR-1986HK, #2225411, Cedarlane Labs, Burlington, Canada) was aliquoted into nuclease-free 0.2 mL PCR tubes (Axygen Scientific PCR-02-C, #10011-780, VWR, Mississauga, Canada) equating to approximately 320 TCID_50_ units (3.6 × 10^5^ RNA genome copies) and stored at −80 °C. An aliquot was added to a saliva-sample tube containing 1 mL 2× stabilization buffer and 1 mL nuclease-free water (in place of saliva), heat-inactivated (10 min at 95 °C), cooled on ice (5 min), and 4 µL was added to the reaction tubes, which was approximately equivalent to 0.64 TCID_50_ units (720 RNA genome copies) of SARS-CoV-2.

## 3. Results

**Figure 3:**
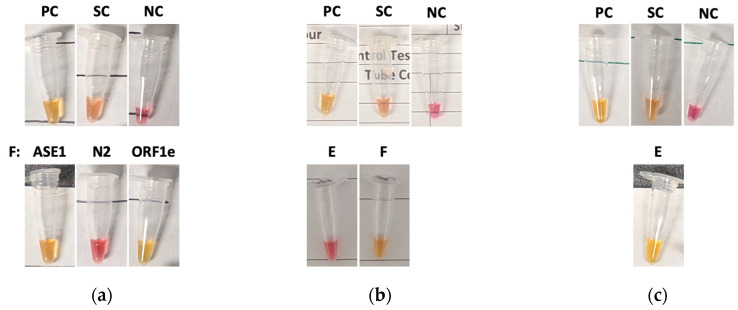
Original RT-LAMP test results from individuals E and F. (**a**) Tuesday, 3 November: individual F’s saliva sample was processed and subjected to RT-LAMP using AS1E, N2, and ORF1e primer sets against SARS-CoV-2. (**b**) Wednesday, 4 November: individual E and F’s saliva were tested using AS1E per a regular scheduled testing day. (**c**) Thursday, 5 November: individual E’s saliva sample was deemed positive with the AS1E primer set against SARS-CoV-2. PC—SARS-CoV-2 virus control; SC—saliva control (RNase P); NC—negative control; AS1E, N2 and ORF1e—primer sets targeting SARS-CoV-2 genes. A negative amplification of the gene target resulted in the solution remaining pink (basic pH), whereas target gene amplification resulted in a color change from pink to yellow (acidic pH).

**Figure 4:**
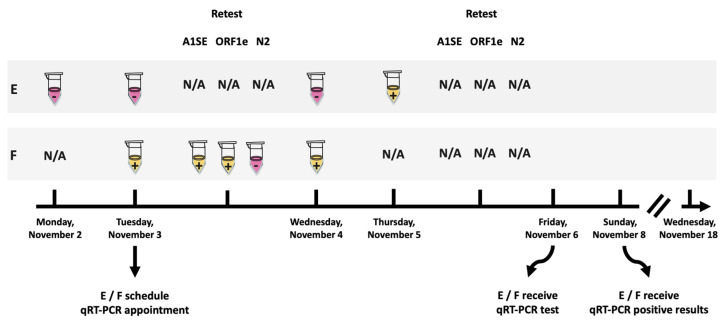
Timeline of E and F cases. Beginning on 2 November, individual E tested negative for SARS-CoV-2. Tuesday, 3 November, saliva samples for E and F were subjected to RT-LAMP using AS1E. Upon F testing positive, the sample retested positive with AS1E and ORF1e, but not for the N2 primer sets against SARS-CoV-2. E and F scheduled an appointment for qRT-PCR testing. On Wednesday, 4 November, E tested negative and F remained positive. On Thursday, 5 November, E’s saliva sample was positive for SARS-CoV-2 with the AS1E primer set and further testing was omitted. Friday, 6 November, and Sunday, 8 November: E and F received qRT-PCR tests and were confirmed positive, respectively. A positive test for SARS-CoV-2 is represented in yellow with a “+” and a negative test is denoted in pink with “-”.

During the 9-month screening program, n = 1649 saliva samples were tested with two positive cases detected—both were from the same household (percent positivity of 0.12%). The epidemiological curve of reported COVID-19 positive cases in Manitoba during the study period is summarized in Figure 1. Two surges in observed cases occurred from 8 November to 28 November, and 9 May to 22 May (Figure 1). During the November case surge, on the evening of Monday, 2 November, an employee, referred herein as “E” (who tested negative earlier that morning on the scheduled testing day) reported that a loved one had a “scratchy throat” (herein referred to as “F”). E was asked not to report for work on Tuesday, 3 November; however, E and F delivered samples contactless for non-scheduled testing. E tested negative, and F tested positive with the AS1E primer set. F was retested with appropriate controls and was found to be positive again with AS1E and with ORF1e, but not with N2 (Figure 3a). Both E and F attempted to isolate from each other at home and immediately scheduled an appointment for qRT-PCR at a COVID-19 testing center. However, as the earliest available was Friday, 6 November, the decision was made that E should not report to work. On the next regularly scheduled testing day (Wednesday, 4 November), E again was negative and F remained positive (Figure 3b). On Thursday, 5 November (non-scheduled testing day), E tested positive with the AS1E primer set and was not retested with any other primer sets. Additionally, F did not provide a sample (Figure 3c). On Friday, 6 November, E and F were administered nasopharyngeal swabs and subsequently tested by qRT-PCR, both were confirmed positive 2 days later. E and F complied with Manitoba Public Health’s recommendations and E returned to work when the isolation period had officially ended on Wednesday, 18 November, as summarized in Figure 4.

## 4. Discussion

We describe a simple and cost-effective program that can be applied in the workplace or other private settings to screen for SARS-CoV-19 infection and to recommend employees or their close contacts for clinical diagnostic testing. The results from screening (around 15 to 20 samples per test day) employees and their friends and family members demonstrate that frequent testing can identify infectious individuals and limit the spread of SARS-CoV-2. Although during the 9-month program only two samples were positive for SARS-CoV-2, public health measures in Manitoba cycled between aggressive lockdowns and slightly relaxed restrictions. Undoubtedly, public health interventions played a role in the decreased case prevalence. In retrospect, given the increasing caseloads, one could have anticipated higher case numbers in the workplace, especially during the epidemiological week of the two positive samples (1 November 2020 to 7 November 2020). The province’s testing and positivity rate increased by 1.1%, a change of n = 1451 administered tests, and n = 1019 more positive cases were observed compared to an earlier incubation period (18–24 October 2020) [15,16]. Moreover, the delays in testing (3 days) and results (2 days) as experienced by E and F could have led to transmission in the workplace and possibly at employee residences or in public settings.

The choice to collect saliva specimens instead of nasal or nasopharyngeal swabs include ease-of-use (self-collection), enhanced safety of testing personnel, and an increased likelihood of compliance. Numerous reports corroborate that the molecular detection of SARS-CoV-2 from saliva samples does not significantly differ from the diagnostic accuracy of nasopharyngeal swabs [17,18,19], especially during the prodromal phase or in asymptomatic individuals [17,20]. A scheme that prioritizes saliva over swabs would be more amenable for routine testing in children. This would be very powerful, as children, despite tending to display milder symptoms or remaining asymptomatic when compared to adults [21], may shed infectious viruses at similar levels [22,23].

Direct saliva testing with the Warmstart product in relation to hydrolysis-probe qRT-PCR, demonstrated a limit of detection (LOD) of 200 virions/µL (Ct of ~30) [10] and 100–1000 viral RNA copies (in vitro transcribed RNA) (Ct of ~30.2 (+/− 2.6)). Thus, Warmstart appears to be 10 to 100 times less sensitive than qRT-PCR [24]. Of note, the sensitivity of RT-LAMP can be improved using alternative enzymes, reagents, dye preparations, and an optional RNA extraction step, as demonstrated after the conclusion of our study [24]. Additionally, RT-LAMP may also be performed using alternative heating sources, including water baths, heating block, or a sous-vide [25,26]. However, a thermometer should be used to confirm temperatures. Differences in sensitivity between RT-LAMP and qRT-PCR have been shown in to be comparable when testing frequency is daily or every three or every seven days in epidemiologic modeling [5]. Thus, the strategy of increased testing frequency can overcome the lower sensitivity with the additional benefits of non-invasive sample collection, lower cost, availability, and consistency when detecting highly contagious individuals (Ct < ~30–32) in real-world settings [10,24]. It is worth noting that these authors used different primer sets for their RT-LAMP studies and that Ct values may vary based on the choice of hydrolysis probes, reagent chemistries, and machine variability.

Variants were not documented by the Manitoba government until the week of 7 February to 13 February 2021 (epidemiological week 6), from then onwards to the end of the study, and the predominant variants were either “undefined” or were B.1.1.7. (Alpha) in approximately 5–35% of cases depending on the week [27]. Using the Global Initiative on Sharing Avian Flu Data (GISAID) initiative, we compared the primer set sequence fidelity (AS1E, ORF1e, and N2) to the first variant described during the study period. In chronological order the Beta, Alpha, Gamma and Delta variants were identified on samples collected on 21 January 2021 (accession: EPI_ISL_1594283), 16 January 2021 (accession: EPI_ISL_1594098), 31 March 2021 (accession: EPI_ISL_1594071), and 29 April 2021 (accession: EPI_ISL_2495627), respectively (Supplementary Table S2). Additionally, the Lambda and Mu variants were not detected prior to 1 June 2021. The AS1E primers remained identical to Alpha, Beta, Gamma, and Delta. However, the F3 from the ORF1e set had a single nucleotide change (C to T) in Alpha only. More importantly, the N2 primer set had two primers, Loop B and FIP, that had multiple changes. Loop B had a single difference (G to T) when compared to Delta, whereas FIP demonstrated 3, 5, and 1 nucleotide changes for Alpha, Gamma, and Delta, respectively (Supplementary Table S2). In the context of F’s saliva sample testing negative with N2, even though Alpha was not documented until January, it is unclear if Alpha may have been circulating during late October. While it is tempting to speculate that an infection with Alpha may have caused this failure, it was more likely a false negative.

The potential for false negative reporting is a limitation of the study: (i) Screening for at least two or two gene targets instead of one (AS1E) would decrease the likelihood of a false negative. (ii) RNaseP detection was only performed in one group per scheduled testing day. It is possible that some saliva samples may be lacking an adequate concentration of buccal cells for each group, indicating poor sample collection.

## 5. Conclusions

During a 9-month voluntary SARS-CoV-2 testing program, two positive samples out of nearly n = 1700 were identified and confirmed by subsequent tests. This type of program helps disseminate the knowledge that inexpensive and convenient primers can be adjusted for new variants for routine rapid testing for SARS-CoV-2. These assays can be accomplished in under an hour with minimal expertise and with reduced risks to the test administrator in multiple settings, including workplaces and schools.

## Data Availability

Manitoba’s epidemiological data can be obtained from the “Epidemiological and Surveillance Unit” provided by the Government of Manitoba (https://www.gov.mb.ca/health/publichealth/surveillance/covid-19/index.html, accessed on 30 November 2021). Positive test case data from routine testing are contained within the manuscript (Figure 1, Figure 3 and Figure 4) and in supplementary material (Tables S1 and S2). Data available upon request due to privacy and ethical restrictions. Official testing result documents presented in this study are available upon request from the corresponding author. The data are not publicly available due to the privacy of the documents.

## Supplementary Materials

The following are available online at https://www.mdpi.com/article/10.3390/diagnostics12020474/s1. Table S1: primer sets for RT-LAMP assay and nucleotide changes observed in variants. Table S2: GISAID sequences used in study.
