# Supplementary material for: Implementation of a Rapid RT-LAMP Saliva-Based SARS-CoV-2 Testing Program in the Workplace
Source: Diagnostics (Basel). 2022 Feb 12;12(2):474. doi: 10.3390/diagnostics12020474 (PMC8871197; doi:10.3390/diagnostics12020474)
Supplement: Supplementary file 1 [file diagnostics-12-00474-s001.zip › diagnostics-1573584-supplementary.pdf]

Supplementary Materials for: Implementation of a Rapid RT-LAMP Saliva-based SARS-CoV-2 Testing Program in the Workplace

Bradley W.M. Cook [1*]., Kaitlyn Kobasa [1,†], Marielou Tamayo [1,†], Natasha Theriault [1,2,†], Diane Gordon Pappas [1], Steven S. Theriault [1,2].

Supplementary Materials

Table S1: Primer Sets For RT-LAMP Assay and Nucleotide Changes Observed in Variants.

Table S2: GISAID Sequences Used in Study.

Supplementary Tables:

Table S1: Primer Sets For RT-LAMP Assay and Nucleotide Changes Observed in Variants.

Primer Set	Primer Name	Primer Sequence (5'-3')	Alpha	Beta	Gamma	Delta
AS1E	F3	CGGTGGACAAATTGTCAC	NC	NC	NC	NC
	B3	CTTCTCTGGATTTAACACACTT	NC	NC	NC	NC
	Loop F	TTACAAGCTTAAAGAATGTCTGAACACT	NC	NC	NC	NC
	Loop B	TTGAATTTAGGTGAAACATTTGTCACG	NC	NC	NC	NC
	FIP	TCAGCACACAAAGCCAAAAATTTATTTTTCTGTGCAAAGGAAATTAAGGAG	NC	NC	NC	NC
	BIP	TATTGGTGGAGCTAAACTTAAAGCCTTTTCTGTACAATCCCTTTGAGTG	NC	NC	NC	NC
N2	F3	CGGCAGTCAAGCCTCTTC	NC	NC	NC	NC
	B3	TTGCTCTCAAGCTGGTTCAA	NC	NC	NC	NC
	Loop F	This primer was omitted from the set. (Yang et al. 2020)	NA	NA	NA	NA
	Loop B	ATGGCGGTGATGCTGCTCTT	NC	NC	NC	1 (G-->T*)<br>ATGGCTGTGATGCTGCTCTT
	FIP	TCCCCTACTGCTGCCTGGAGCGTTCCTCATCACGTAGTCG	3 (CCC-->GTT*)<br>TCGTTTACTGCCTGGAGCGTTCCTCATCACGTAGTCG	NC	5 (CCC -->GTT*, CT-->GA*)<br>TCGTTTAGAGCTGCCTGGAGCGTTCCTCATCACGTAGTCG	1 (C-->A*)<br>TCCCATACTGCTGCCTGGAGCGTTCCTCATCACGTAGTCG
	BIP	TCTCCTGCTAGAATGGCTGGCATCTGTCAAGCAGCAGCAAAG	NC	NC	NC	NC
ORF1e	F3	GGCTAACTAACATCTTTGGC	1 (C-->T*)<br>GGCTAACTAATATCTTTGGC	NC	NC	NC
	B3	GTCAGCACACAAAGCCAA	NC	NC	NC	NC
	Loop F	TCTTCAAGCCAATCAAGGAC	NC	NC	NC	NC
	Loop B	TTGTCGGTGGACAAATTGT	NC	NC	NC	NC
	FIP	TCTCTAAGAAACTCTACACCTTCCTTTTTACTGTTTATGAAAAACTCAAACC	NC	NC	NC	NC
	BIP	TATCTCAACCTGTGCTTGTGAAATTTTAGAATGTCTGAACACTCTCCT	NC	NC	NC	NC
RNase P	F3	TTGATGAGCTGGAGCCA				
	B3	CACCCTCAATGCAGAGTC				
	Loop F	ATGTGGATGGCTGAGTTGTT				
	Loop B	CATGCTGAGTACTGGACCTC				

	FIP	GTGTGACCCTGAAGACTCGGTTTT AGCCACTGACTCGGATC	

* Change observed in variant

Supplementary Tables:

Table S2: GISAID Sequences Used in Study.

Variant	Isolate	Accession ID	Collection Date	Submission Date
Alpha	hCoV-19/Canada/MB-NML-16346/2021	EPI_ISL_1594283	2021-01-21	2021-04-15
Beta	hCoV-19/Canada/MB-NML-17441/2021	EPI_ISL_1594098	2021-01-16	2021-04-15
Gamma	hCoV-19/Canada/MB-NML-34961/2021	EPI_ISL_1594071	2021-03-31	2021-04-15
Delta	hCoV-19/Canada/MB-NML-70550/2021	EPI_ISL_2495627	2021-04-29	2021-06-11

All samples were collected from MB-Cadham Laboratory and submitted by the National Microbiology Laboratory (NML) in North America, Canada, Manitoba by: Anna Majer; Anneliese Landgraff; CanCOGeN's metadata curation team; Darian Hole; David Alexander; Elsie Grudeski; Gary Van Domselaar; Grace Seo; Jared Bullard; Jennifer Tanner; Kerry Dust; Kirsten Biggar; Madison Chapel; Morag Graham; Natalie Knox; Nathalie Bastien; Paul Van Caeseele; Philip Mabon; Public Health Agency of Canada CanCOGeN team; Rhiannon Huzarewich; Russell Mandes; Shari Tyson; Timothy Booth; Yan Li.
